# Errors in Table, Text, and References

**DOI:** 10.1001/jamahealthforum.2023.1834

**Published:** 2023-06-16

The Viewpoint titled “Cautions When Using Race and Ethnicity in Administrative Claims Data Sets,”^1^ published on July 1, 2022, was corrected to fix errors in the table, text, and references related to information regarding Optum Clinformatics Data Mart. A reference was removed, and the table and article text were edited. This article was corrected online.
